# Welfare impact of climate change on capture fisheries in Vietnam

**DOI:** 10.1371/journal.pone.0264997

**Published:** 2022-04-25

**Authors:** Thi Vinh Ha Nguyen

**Affiliations:** University of Economics and Business, Vietnam National University, Hanoi, Vietnam; Duy Tan University, VIET NAM

## Abstract

Fisheries are forecasted to shrink in the tropics due to climate change. In Vietnam, fisheries are a pro-poor economic sector and essential nutrition source; however, welfares of producers and consumers in the climate change context are not well understood. While most studies focus on the gains or losses of total products and revenues, this paper pays additional attention to the changes in surpluses of market players in the long run. A combination of the production function, demand and supply functions, and partial equilibrium analysis is employed to measure the production and welfare impacts based on time series data from 1976 to 2018 and a Vietnam household living standards survey in 2018. The results show that relative to the present, catch yield is likely to reduce 35%-45% by mid-century and 45%-80% by the end of the century. Consumers may lose their surplus of 7-9 billion USD (PPP, 2018) by 2035 and 10-18 billion USD by 2065 due to supply reduction, while producers may gain additional profit of 3.5-4.5 billion USD by 2035 and 5-9 billion USD by 2065 owing to a price increase. The research findings suggest that Vietnam could impose measures to limit capture effort, as set out in the Law of Fisheries 2017, without harming fisher welfare. The expansion of aquaculture could reduce the gap between supply and demand of wild fish to mitigate consumer welfare loss; however, this impact is still ambiguous.

## Introduction

Vietnam is a coastal country on the west bank of the East Sea, with a coastline of 3,444 km stretching from North to South and more than 3,000 large and small islands [1]. There are ample fish resources along the coast, allowing Vietnam to rank seventh in the major marine capture countries and territories. The total marine catch reached 3.2 million tons, accounting for 4% of the world’s production in 2018 [2]. Fisheries is Vietnam’s key economic sector, contributing 23.75% to agricultural products and 3.43% to gross domestic products [3].

Climate change impacts are witnessed in various aspects of human life and living organisms around the globe, in which fisheries are widely recognized to be affected [4–7]. Climate change directly disturbs aqua species’ growth, reproduction, migration behavior, and distribution, as it induces fluctuations in physical, chemical, and biological factors [8–12]. Indirect impacts via ecosystems include changes in food abundance, competitors, predators, and pathogens [13–16]. Climate change leads to loss of catch revenues and profits of entrepreneurs and households in many areas, especially in countries with warm seas. It sometimes benefits other countries, such as those with cold water [17–22]. However, fisheries in both regions might get damaged due to water quality degradation and the spread of diseases [23–25].

Several studies have quantified the impacts of climate change on fisheries, revealing different magnitudes in different places and species. The catch potential of Indian mackerel for the period 2020-2100 under RCP6.0 will decline from 2035 (+14%) to 2100 (−44%) [26]. The predicted loss of the fishery sector in Guyana under A2 and B2 scenarios in 2050 are from 20 to 34 million USD at the discount rate of 1% per year [27]. Dey, Gosh [28] report that total fish demand will likely surpass domestic fish production in 2050 in Fiji due to the declining trend of fish capture in climate change scenarios. The global economic costs of mollusk due to ocean acidification could be over 100 billion USD with an assumption of increasing demand of mollusks at expected income growths and a business-as-usual emission trend towards the year 2100 [29].

Cheung, Lam [30] forecast a drop of up to 40% catch potential due to climate change for 2005-2055 in the tropics. Since Vietnam is located in a tropical region, climate change is likely to affect its fishery sector adversely. Lam, Cheung [22] estimate change in global fishery revenues under climate change and estimate a reduction of 11.3% between 2010 and 2050 in Vietnam. The Vietnam Fisheries Development Strategy for 2021-2030 [31] projects a shrink in catch yield from 3.8 million tons in 2020 to 2.8 million tons in 2030.

Fisheries are a pro-poor economic sector and important nutrition source in Vietnam [31]. This sector provides jobs to 3 million laborers, accounting for 17% of total laborers in agriculture and 5.6% in the national labor force [3]. Fishery products meet 40% of food consumption demand, contributing significantly to ensuring national food security and nutrition requirements [32]. Climate change seems to influence both fishery laborers and consumers.

Reviews of the literature reveal that most studies related to impacts of climate change focus on the gains or losses of fish stocks, yields and revenues, while the welfare of fishery producers and consumers under climate change is not well understood. When the supply falls short of demand, the price will increase, leading to the uncertain fluctuation in welfares of market players. The study aims to identify the welfare changes via a partial equilibrium analysis and supporting methods. It is the first attempt to quantify the losses, or gains, if it is the case, of wild fish consumers, producers, and both due to climate change in Vietnam.

## Conceptual framework

There are several approaches to analyze the various aspects of climate change impacts, such as vulnerability [5, 33–36], adaptation and mitigation [6, 37–41], and economic impact [27, 28, 42–46]. Since welfare is the primary concern of this study, its approach should fall in the range of economic issues. Hence, we follow Brander [4] and Sumaila [7] to build the conceptual framework, as it allows us to consider the impact of climate change on fishery productions, costs, revenues and incomes.

**Fig 1:**
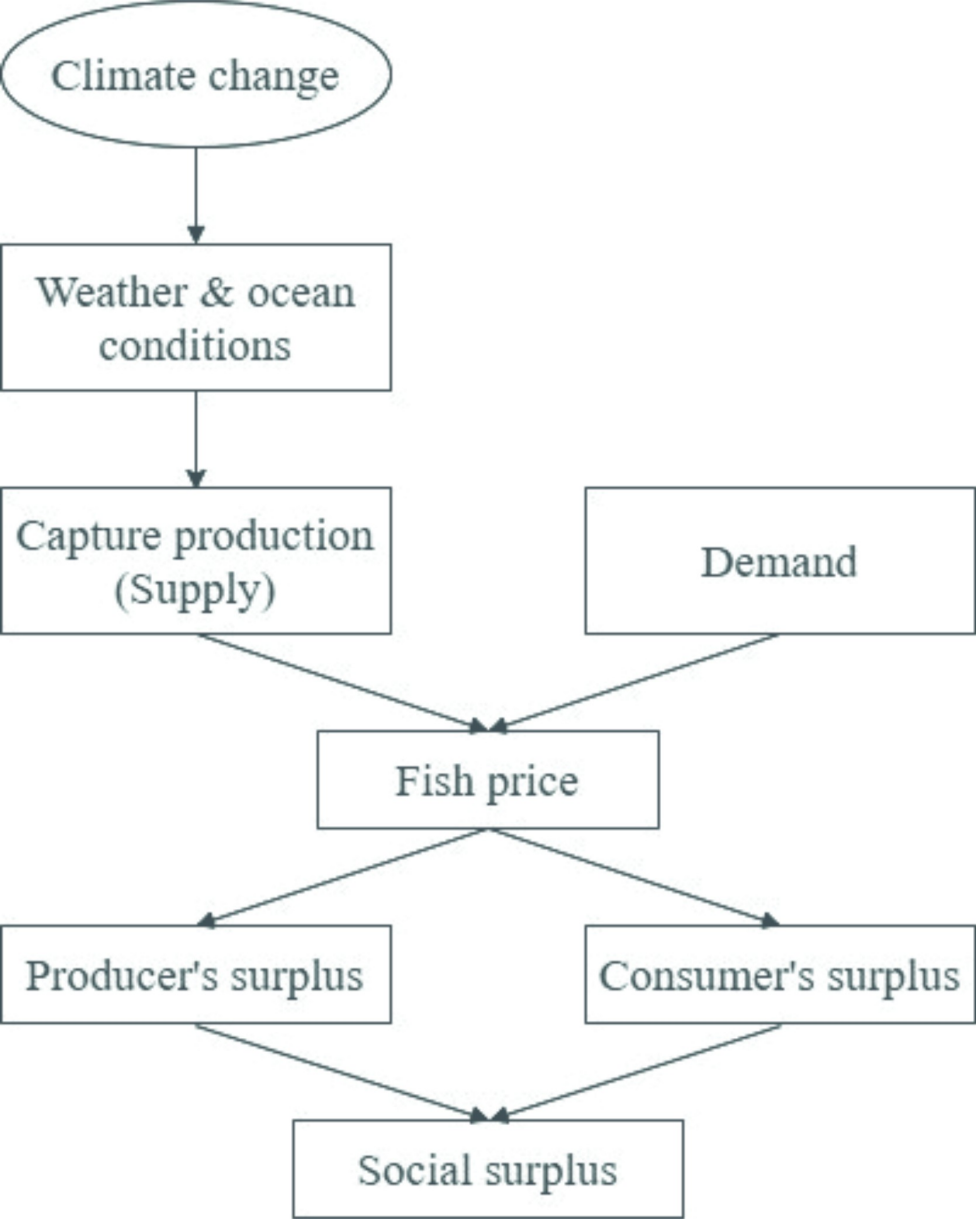
The welfare impact of climate change on fishery consumers and producers.

There is ample literature depicting the impacts of climate change on aqua species [2, 4, 7, 17, 24], which includes instabilities in physical and biological ecosystems, primary and secondary productivity, and fish stock distribution. These volatilities then affect fishing costs, prices, resource rents, revenues, and profits [4, 7, 47]. Fig 1 describes the flow of climate change impacts on the welfares of fishery consumers and producers.

As shown in Fig 1, the sequence of impacts starts from climate change, which is an irreversible trend in the 21^st^ century [48]. Its manifestations include changes in weather and ocean conditions: higher sea surface temperature in most oceans, the most significant change is in the tropics and subtropics of the Northern hemisphere [48, 49]; changes in ocean currents and wind regimes [48]; more intensive annual rainfall in high latitude and equatorial Pacific regions [50], which leads to varying salinity and rising acidity [51, 52]; increase in the frequency of extreme El Niño and La Niña events [53].

Marine fishes and other aquatic species are poikilotherms whose internal temperature varies considerably. They are susceptible to variation in environmental temperature and migrate to water areas with their preferred warmth [23, 54–56]. Lower oxygen concentration and higher hydrogen potential alter unfavorably to aquatic species [57, 58]. Acidification is also a reason for widespread coral bleaching, harming aquatic habitats [59]. In response, tropic fish may reduce their size by age for lower metabolism [4, 7]. Precipitation affects species’ growth by changing the water environment, such as salinity and contamination [60]. Typhoons keep fishers away from production, damage vessels [61], and increase marine contamination [62]. Southern Oscillation, including El Niño and La Niña, may alter fish flows and affect total catches [63, 64]. Heavy typhoons and tornados destroy coral reefs [48]. Changes in climate and marine conditions indirectly influence species, increasing the sensitivity of planktons, invertebrates, crustaceans, food web, and diseases [17, 59].

Fishing costs may increase due to incremental investment in vessels and gears to adapt to changes in fish stocks, species composition, and distribution [7]. Changes in migration behavior and fish stock distribution may lengthen the travel time of fishing boats, escalating fuel and ice consumption. A decrease in fish stocks and an increase in fishing costs will reduce production and shift the supply curve to the left side.

On the demand side, total wild fish consumption depends on several factors such as population size, income, preferences, and the availability of substitutes, especially aquacultural products. The relative fishery price is likely to go up when the potential catch goes down in the long run.

Profit will alter following changes in catch yields, costs, and prices. The welfare benefits of consumers, producers, and society will be reallocated.

Following the flow of climate change impact on fisheries, this paper tests and measures (1) the capture reduction due to climate change, and (2) the potential changes in producer and consumer welfares.

## Research methods and data

This study employs a set of several methods. Impact on catch yields is forecasted using a production function and the climate change scenarios for Vietnam. Then, a partial equilibrium analysis measures welfare changes. The price elasticity of demand is identified via a Marshallian demand function. The price elasticity of supply is inherited from the IMPACT model of IFPRI [29]. Different data sets are used for different methods.

### Production function

The production function is widely used in literature to describe the relationship between outputs and inputs in agriculture and fisheries [42, 47, 65–68]. Following Conrad [69], the Cobb-Douglas function is served as a base to develop the projection model. Two main production inputs are labor and capital; the latter is proxied by total fleet gears [70]. Climate change indicators such as sea surface temperature, precipitation, number of typhoons, maximum wind speeds of typhoons, and Southern Oscillation index are assumed to impact total catches. A dummy reflects the effectiveness of the Law on Fisheries, which was first introduced in 2003.

The production function to evaluate impacts of climate change on capture fisheries in Vietnam is formed as Eq (1):

$$LnCatch_{t}=\beta_{0}+\beta_{1}T+\beta_{2}LnCapacity_{t}+\beta_{3}LnLabor_{t}+\beta_{4}SST_{t}+\beta_{5}LnRainfall_{t}+\beta_{6}Typhoon_{t}+\beta_{7}LnWindmax_{t}+\beta_{8}SOI_{t}+\beta_{9}D+\varepsilon_{t}$$ (1)

where *β*_*i*_ and *ε*_*t*_ are coefficients and white noise, respectively.

The North and the South of Vietnam were united in 1975. Therefore, the time-series data can only be collected uniformly from 1976 until recently, i.e., 2018. Annual data is employed due to the seasonal characteristics of capture production. Data are mined from their most reliable sources: data on catch yield, capacity, and labors are collected from the Vietnam General Statistical Office; climate data such as sea surface temperature, rainfall, and Southern Oscillation index are exploited from the National Oceanic and Atmospheric Administration (USA) database and the World Bank climate knowledge portal; data on typhoons and their maximum wind speeds are from the Joint Typhoon Warning Center (USA) database. Variable descriptions and sources of data are described in Table 1.

**Table 1:** Variable descriptions and sources.

Variable	Description	Sources of indicator	Sources of data	Mean	Minimum	Maximum	Std. Dev.
*Catch*	Total catches (tons)	Alnafissa, Kotb [47] Ibarra, Armando [42] Sun, Chiang [71]	Vietnam Institute of Fisheries Economics and Planning -VIFEP [72] gso.gov.vn	1,406,827	1,078,630	3,189,889	907,366
*Capacity*	Total vessel gears (CV)	Ibarra, Armando [42] Kishida, Wada [70]	VIFEP [72] gso.gov.vn	3,725,295	453,871	14,480,600	3,994,537
*Labor*	Number of fishers (persons)	Alnafissa, Kotb [47] Ibarra, Armando [42]	VIFEP [72] FAO [2]	913,235	190,339	2,186,850	733,051
*SST*_*t*_	Annual average sea surface temperature (°C) (108E, 18N)	Alnafissa, Kotb [47] Ibarra, Armando [42] Meynecke, Grubert [64]	ncdc.noaa.gov	26.16	25.41	26.89	0.37
*Rainfall*	Total annual precipitation (mm)	Meynecke, Grubert [64] Tseng and Chen [73]	climateknowledgeportal.worldbank.org	1,822	1,536	2,212	153
*SOI*	Annual Southern Oscillation index	Meynecke, Grubert [64] Sun, Chiang [71]	ncdc.noaa.gov	0.04	-1.53	2.30	0.96
*Typhoon*	Number of typhoons on the East Sea, from mainland up to 120E and 10N	Monteclaro, Quinitio [62]	nchmf.gov.vn usno.navy.mil/JTWC	8.09	2	18	3.36
*Windmax*	Maximum wind speed of typhoons	Alnafissa, Kotb [47]	nchmf.gov.vn usno.navy.mil/JTWC	170.70	100	230	32.12
*t*	Year	Sun, Chiang [71]	From 1976 to 2018				

The Autoregressive-distributed lag (ARDL) model is applied to capture the lag impacts of the previous year(s) on total catches. ARDL is appropriate for regression with few observations [74]. The long-run form derived from ARDL regression is employed to estimate the long-run impact. Several tests are conducted to assess the properness and reliability of the ARDL model [74, 75].

Vietnam’s climate change scenarios developed by the Ministry of Natural Resources and Environment in 2016 [76] and updated in 2021 [77] are used for forecasting the impact of climate change in the early, mid and end periods of the 21^st^ century, i.e., 2018-2035, 2046-2065, and 2080-2099, based on the RCPs 4.5 and 8.5 for 32 coastal provinces of Vietnam.

### Partial equilibrium analysis

There are two major types of supply-demand analysis: general equilibrium and partial equilibrium. In the general equilibrium analysis, the market system includes many separate but connected markets, of which the fishery market is one component. In the market system, all goods and factors of production are interrelated so that prices in all markets are set at the same time. Partial equilibrium analysis is limited to the study of supply-demand balance in individual markets (e.g., rice market, flower market). The primary feature of partial equilibrium analysis is the use of supply and demand curves of a particular commodity to determine its prices and quantities.

This study focuses on the welfare of fishery consumers and producers. Hence, we have chosen the partial equilibrium analysis [68] to evaluate producer and consumer surplus changes based on neoclassical welfare economics. For the simplicity of analysis, it is acceptable to assume that the supply and demand curves have constant price elasticities. Then the demand and supply functions have the following forms:

$$Q_{D}=A_{D}P^{\varepsilon_{D}}\;\mathrm{or}\;\ln\;Q_{D}=\ln\;A_{D}+\varepsilon_{D}\;\ln\;P$$ (2)

$$Q_{S}=A_{S}P^{\varepsilon_{S}}\;\mathrm{or}\;\ln\;Q_{S}=\ln\;A_{S}+\varepsilon_{S}\;\ln\;P$$ (3)

in which *P* is fish price; *Q*_*D*_ and *Q*_*S*_ are demand and supply quantities, respectively; *A*_*D*_ and *A*_*S*_ are constants; and *ε*_*D*_ and *ε*_*S*_ are price elasticities of demand and supply, respectively. Since the demand curve slopes downward and the supply curve slopes upward, we have *ε*_*D*_ < 0 and *ε*_*S*_ > 0.

At the initial state, the price equilibrium is determined at

$$Q_{D_{0}}=Q_{S_{0}}$$ (4)

When the relative changes in demand and supply are *a* and *b*, respectively (*a*, *b* > 1 if the change is an increase; 0 ≤ *a*, *b* < 1 if the change is a decrease; *a = 1* or *b* = 1 if there is no change), the new supply and demand functions are as follows:

$$Q_{D_{1}}=aQ_{D_{0}}$$ (5)

$$Q_{S_{1}}=bQ_{S_{0}}$$ (6)

The new equilibrium price is identified at

$$Q_{D_{1}}=Q_{S_{1}}$$ (7)

Solve the system of Eqs from (2) to (7); we arrive at the relative price change:

$$\frac{P_{1}}{P_{0}}={\left(\frac{a}{b}\right)}^{\frac{1}{\varepsilon_{S}-\varepsilon_{D}}}$$ (8)

Change in consumer surplus:

$$\begin{array}{l} \Delta CS=\int_{P_{1}}^{P_{max}}{aA}_{D}P^{\varepsilon_{D}}-\int_{P_{0}}^{P_{max}}A_{D}P^{\varepsilon_{D}}\\ \;=\frac{A_{D}}{1+\varepsilon_{D}}(P_{0}^{1+\varepsilon_{D}}{-aP}_{1}^{1+\varepsilon_{D}})+\frac{(a-1)A_{D}}{1+\varepsilon_{D}}P_{max}^{{1+\varepsilon }_{D}} \end{array}$$ (9)

$$\frac{{CS}_{1}}{{CS}_{0}}=\frac{\frac{aA_{D}}{1+\varepsilon_{D}}(P_{max}^{1+\varepsilon_{D}}{-P}_{1}^{1+\varepsilon_{D}})}{\frac{A_{D}}{1+\varepsilon_{D}}(P_{max}^{1+\varepsilon_{D}}{-P}_{0}^{1+\varepsilon_{D}})}=a\left(1-\frac{P_{1}^{1+\varepsilon_{D}}{-P}_{0}^{1+\varepsilon_{D}}}{P_{max}^{1+\varepsilon_{D}}{-P}_{0}^{1+\varepsilon_{D}}}\right)$$ (10)

Change in producer surplus:

$$\begin{array}{l} \Delta PS=\int_{P_{min}}^{P_{1}}{bA}_{S}P^{\varepsilon_{S}}-\int_{P_{min}}^{P_{0}}A_{S}P^{\varepsilon_{S}}\\ \;=\frac{A_{S}}{1+\varepsilon_{S}}(bP_{1}^{1+\varepsilon_{S}}–P_{0}^{1+\varepsilon_{S}}) \end{array}$$ (11)

$$\frac{{PS}_{1}}{{PS}_{0}}=a^{\frac{\left(1+\varepsilon_{S}\right)}{\varepsilon_{S}-\varepsilon_{D}}}b^{\frac{\left(-1-\varepsilon_{D}\right)}{\varepsilon_{S}-\varepsilon_{D}}}$$ (12)

Change in social surplus:

ΔSS=ΔCS+ΔPS (13)

### Demand function

About 85% of Vietnam’s total catches are for domestic use, and the rest, 15%, is for export [2]. It is acceptable to assume that the own-price elasticity of total demand for fisheries equals its domestic one. Total market demand, by economic principles, is the sum of individual household demands. Therefore, we assume that the individual households and the total market for wild fish bear identical demand elasticities.

We recall the demand function from Eq (2):

$$\ln\;Q_{D}=\ln\;A_{D}+\varepsilon_{D}\;ln\;P$$ (2)

In microeconomics, the consumer’s Marshallian demand depends on its price, consumer income, and prices of substitutes. Household demand also varies on household size, number of children, and other factors such as region, urban/rural location, occupation, employment, education, age, race, religion, crop, and availability of fishery shops [78].

Data from Vietnam Household Living Standards Survey (VHLSS) in 2018 [79] are exploited to develop the household demand function. The survey covered 9,399 households, representative at national, regional, urban, rural, and provincial levels. The survey collected information on household’s income, expenditure and other issues during four periods, each period in one quarter from the first to the fourth quarter in 2018 through face-to-face interviews conducted by interviewers with household heads and household members. After omitting missing values, the final sample for the demand function regression model has 8,288 observations.

The dependent variable is fishery consumption quantity in 12 months in logarithm form, irrespective of capture or culture. In Vietnam, substitutes for fishery products are often pork and chicken. Therefore, the fishery demand function has the following form:

$$\ln Q\mathrm{fish}=\beta_{0}+\beta_{1}\;\ln P\mathrm{fish}+\beta_{2}\;\ln Y+\beta_{3}\;\ln\mathrm{Ppork}+\beta_{4}\;\ln\mathrm{Pchicken}+\sum_{i=5}^{n}\beta_{i}X_{i}+\varepsilon_{t}$$ (14)

whereas

*Qfish* is household fish intake in 12 months (kg);

*Pfish* is fish price paid by household (thousand dongs per kg);

*Y* is the household income per capita (thousand dongs);

*Ppork* and *Pchicken* are prices of substitutes, which are pork and chicken (thousand dongs per kg);

*X*_*i*_ is a set of other independent variables. Following Cheng and Capps [78], *X*_*i*_ include dummies for five geographical regions: Red River Delta, North Central and Coastal Central, Central Highland, Southeast, and Mekong Delta (Northwest region is the base category); coastal provinces, number of household members, and characteristics of household head such as gender, age, education, marital status, and occupation (self-employed in agriculture, self-employed in non-agriculture or service sector; non-self-employment is the base category);

*β*_*k*_ are coefficients, in which *β*_*1*_ is the own-price elasticity, and *ε*_*t*_ is the error term.

## Results

### Impact of climate change on potential catches

The long-run form of the ARDL model indicates the long-run impact of climate change on catch fisheries. The results are presented in Table 2.

**Table 2:** Long-run form of the production function.

*Variable*	*Coefficient*		*Standard Error*	*ADF Statistic*			*Number of lags in ARDL model*
Ln*Capacity*	0.1453	**	0.0567	-3.8608	**	I(0)	2
Ln*Labor*	-0.1180		0.0756	-2.6976	*	I(1)	2
SST	-0.1961	***	0.0516	-3.7521	***	I(0)	3
Ln*Rainfall*	-0.4372	**	0.1421	-5.6733	***	I(0)	3
Typhoon	-0.0276	***	0.0063	-5.2423	***	I(0)	3
Ln*Windmax*	-0.1806	*	0.0896	-5.4866	***	I(0)	3
SOI	0.0623	***	0.0101	-4.5769	***	I(0)	3
Number of observations after adjustment				40			
Number of lags of dependent variable Ln*Catch*				3			
ADF statistic of dependent variable Ln*Catch*, I(0)				-4.0360	**		
ADF statistic of residuals, I(0)				-7.6431	***		
Adjusted *R*^2^ (Error Correction Model)				0.9588			
CointEq(-1)* coefficient (Error Correction Model)				-1.1449	***		
Durbin-Watson statistic				2.2006			
F-bound test: F-Statistic (*k* = 7)				29.2657	***		
t-bound test: t-statistic				-10.8023	***		
Breusch-Godfrey Serial Correlation LM Test: *nR*^2^				1.8385	^NS^		
Breusch-Pagan-Godfrey Heteroskedasticity Test: *nR*^2^ (*df* = 31)				36.5049	^NS^		
Jarque-Bera Statistic of residuals (normality test)				0.1278	^NS^		
Ramsey RESET test: t-statistic (*df* = 7)				0.9089	^NS^		
F-statistic *df* = (1,7)				0.8261	^NS^		

*p < 0.1

**p < 0.05

***p < 0.01

^NS^ Non-significant

All the statistics for model assessment show that the regression model is appropriate, and the results are statistically reliable.

The regression results (Table 2) show that total vessel gears play a critical role in determining catch yield while the number of laborers is not significant. When sea surface temperature increases by 1°C, the total catches fall 19.61%. If precipitation rises 1%, the output drops 0.44%. An additional typhoon in a year decreases the capture yield by 2.76%. The severity of storms also negatively influences output. When the maximum wind speed of typhoons increases by 1%, the yield reduces by 0.18%. The coefficient of *SOI* is positive, indicating that catch yield decreases when El Niño occurs and increases in the case of La Niña. However, the long-term impact of SOI is neglectable, as 1 point decrease in SOI leads to a merely 0.06% decrease in capture yield.

IPCC [48] reports that the changing trend in the number of typhoons is not evident during the past decades. Vietnam’s climate change scenarios, developed by the Ministry of Natural Resources and Environment [77], indicate that there might be a decrease in frequency in the number of typhoons by the end of the 21^st^ century under the RCP8.5. Under the RCP4.5, the typhoon frequency is not likely to change. Therefore, we will not consider the typhoon frequency and Southern Oscillation to pay attention to assess the impact of change in temperature, rainfall and maximum wind speed of typhoons on capture fisheries in the long run.

Table 3 summarizes the projected change in different scenarios compared to 2018. Changes in temperature and precipitation in early, mid and end periods of the 21^st^ century, i.e., 2018-2035, 2046-2065, and 2080-2099, are estimated based on the RCPs 4.5 and 8.5 for coastal provinces of Vietnam [76, 77].

**Table 3:** Changes in temperature, precipitation, and maximum wind speed of typhoons in early, mid and end periods of the 21^st^ century in Vietnam relative to 2018.

Average change in	RCP4.5			RCP8.5		
	2018-2035	2046-2065	2080-2099	2018-2035	2046-2065	2080-2099
Temperature (°C) (lower 10% – upper 90%)	0.7 (0.4–1.2)	1.4 (0.9–2.2)	1.8 (1.2–2.6)	0.9 (0.6–1.3)	1.9 (1.3–2.6)	3.4 (2.6–4.5)
Precipitation (%) (lower 20% – upper 80%)	13.2 (1.4–24)	14.9 (1.6–28.7)	17.2 (4.1–30.5)	13.5 (4 – 24.5)	17.1 (7.3–27)	21.3 (8.5–35.5)
Maximum wind speed (%)	2.0	5.0	8.0	2.0	6.0	11.0

Values in parentheses are confident intervals: lower 10% – upper 90% for temperature; and lower 20% – upper 80% for precipitation.

Source: Based on MONRE [76, 77]

As shown in Table 3, the temperature will rise in both RCPs. 4.5 and 8.5, from 1.4 to 1.9°C by mid-century (2046-2065) and from 1.8 to 3.4°C by the end of the century (2080-2099). Rainfall may increase or decrease in different areas in different scenarios, but precipitation will average from 15% to 17% by mid-century and 17% to 21% by end-century. The typhoon intensity is likely to increase from 2% to 11% [77].

**Fig 2:**
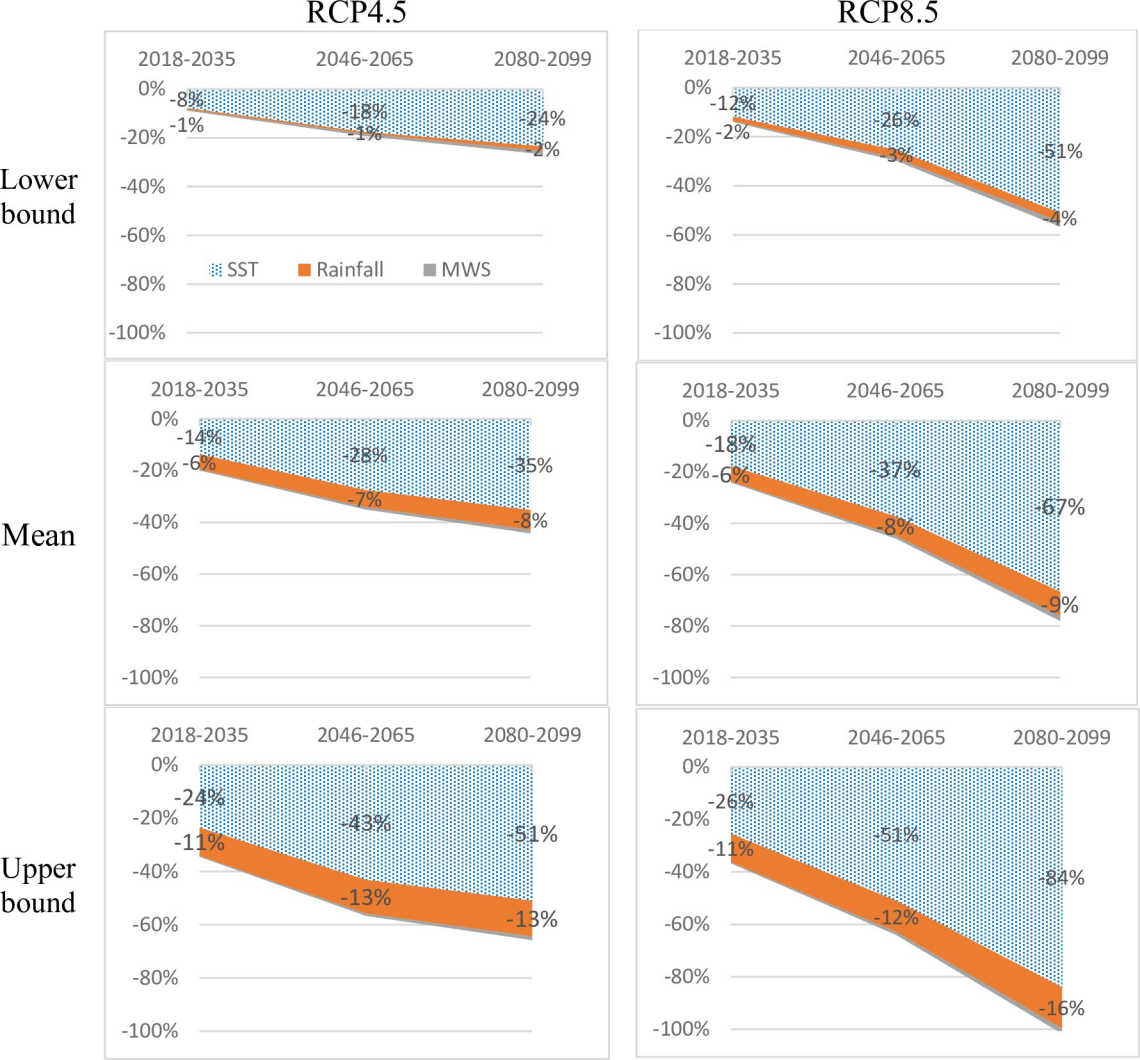
Potential decrease in capture yield in early, mid and end of the 21^st^ century by climate change scenarios RCP4.5 and RCP8.5 relative to 2018.

Fig 2 illustrates the potential decrease in catch yield compared to 2018, based on the RCPs 4.5 and 8.5.

Fig 2 shows that temperature rise accounts for 70% to 90% of the catch reduction, averaging 27.5% to 37.3% by mid-century, 35.3% to 66.7% by end-century relative to 2018, depending on climate change scenarios. Precipitation has a considerable impact if the temperature projection falls from average to upper bound, at 6.6% to 7.5% by mid-century and 7.6% to 9.4% by end-century. At the lower bound, the impact of rainfall is relatively tiny. The impact of typhoon maximum wind speed is almost insignificant, approximately 1% of the total catch in 2018.

**Fig 3:**
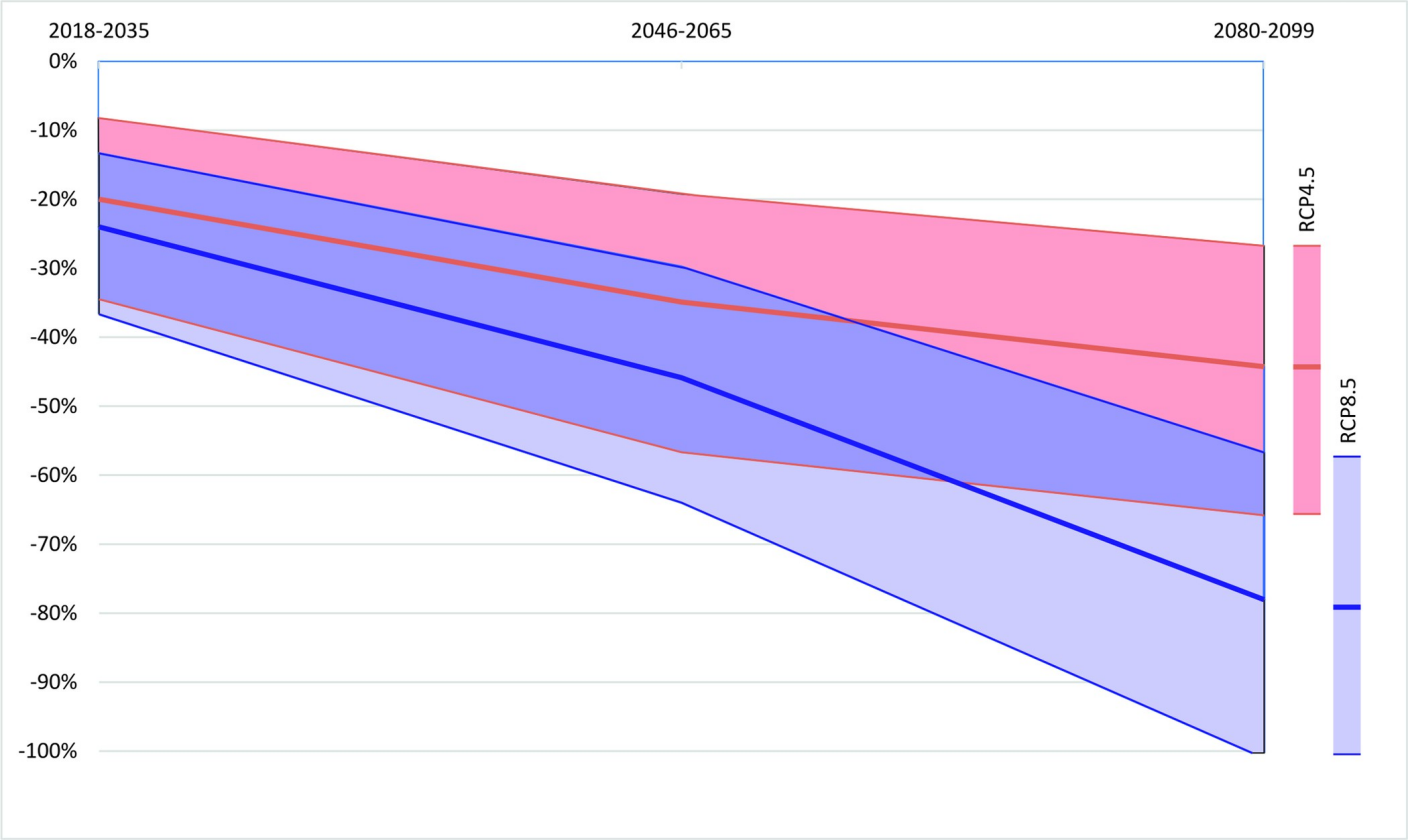
Potential decrease in capture yield due to climate change relative to 2018.

Fig 3 sums up the impact of temperature, precipitation and maximum wind speed on catch yield at lower, mean and upper bounds of each climate change scenario.

Fig 3 illustrates that, under the RCP4.5, the capture yield will reduce by 35% from now to mid-century, then it will continue to shrink at a slower speed until the end of the century, relative to 44% of the total catch in 2018. The contraction under RCP8.5 almost doubles the estimates under RCP4.5, at 46% by mid-century and 78% by end-century. There might be almost no marine capture by the end of the century in the worst case.

### Price elasticities of demand and supply on fishery market

Table 4 presents the demand function regression results using VHLSS data in 2018. 

**Table 4:** Household’s fishery demand function in Vietnam, 2018.

*Variable*	*Coefficient*		*Standard Error*
Fish price (log)	-0.1618	***	0.0158
Income (log)	0.1263	***	0.0107
Pork price (log)	0.2693	***	0.0377
Chicken price (log)	0.0683	***	0.0179
Household size	0.1405	***	0.0061
Red River Delta	0.2964	***	0.0237
North Central Coast	0.3296	***	0.0409
Central Highlands	0.2899	***	0.0303
Southeast	0.5510	***	0.0311
Mekong River Delta	0.8005	***	0.0354
Coastal provinces	0.3706	***	0.0312
Gender of household head	-0.4462	***	0.0191
Age of household head	0.0021	***	0.0006
Marital status household head	0.3723	***	0.0225
Years of schooling of household head	-0.0206	***	0.0018
Agricultural jobs	0.0510	***	0.0195
Service jobs	0.0101		0.0197
_cons	-0.5427	***	0.1984

Weighted Least Square Regression: *Dependent variable*: *Fish consumption quantity (log)*

*n* = 8288, *Prob(F)* = 0.0000, Adjusted *R*^2^ = 0.4395

*p < 0.1

**p < 0.05

***p < 0.01

The regression results (Table 4) indicate that the fishery demand depends negatively on its price and positively on the prices of substitutes such as pork and chicken. The fishery demand also depends on income per capita, household size, and other household characteristics. The demand for fishery products is higher in regions with more fish availability, ranking from Mekong River Delta, Southeast, North Central Coast, Red River Delta, Central Highlands, and the lowest demand is in Northern Mountains. The demand in coastal provinces is higher than in the inland. This result proves that fish consumption preference is likely to reduce when total catch decreases.

The own-price elasticity of fishery demand is *ɛ*_D_ = -0.16, i.e., when the price goes up by 1%, the demand drops by 0.16%. Similar elasticities are obtained when data from VHLSS in 2012 and 2014 are used [80], indicating that the own-price elasticity of fisheries stands still over time. Since Vietnam is located along the seaside, artisanal fishing is widespread; fishery products are popular and essential in the daily food consumption of households. These factors explain the steep elasticity of fishery demand in Vietnam compared to China [81] or the United States [78]. However, it is flatter than in Indonesia [82], where people live on islands and rely more on fish for nutrition.

The International Model for Policy Analysis of Agricultural Commodities and Trade (IMPACT) of the IFPRI [29] uses a system of price elasticities of fisheries and 22 non-fisheries commodities in 36 regions and countries, including Southeast Asia, to estimate fishery supply and demand functions. It finds that capture fishery price elasticity of supply is between 0.2 and 0.4 worldwide. This study chooses *ɛ*_S_ = 0.2, as capture in Vietnam is over the maximum sustainable yield [72]; consequently, the supply curve tends to be steeper than average. When the price goes up by 1%, its supply grows by 0.2%; other things are equal.

### Welfare impact of climate change on consumers and producers

Replacing the total catches *Q*_D_ = *Q*_S0_ = 3.2 million tons, the average fish price *P*_0_ = 24.5 million dongs per ton in 2018 [2], and the price elasticities *ε*_*D*_ = -0.16 and *ε*_*S*_ = 0.20 into the demand and supply functions (2) and (3), we obtain *A*_D_ = 6.0 and *A*_S_ = 1.4.

Based on the Eqs (6–9), the changes in social welfares by 2035 and 2065 are calculated. Since the potential catch is negatively affected by climate change, in Eq (6), we have 0 < *b* < 1. The value of *b* is estimated as followings:

By 2035, *b*∈(0.66, 0.91) for RCP4.5; *b*∈(0.63, 0.86) for RCP8.5;

By 2065, *b*∈(0.43, 0.81) for RCP4.5; *b*∈(0.36, 0.70) for RCP8.5.

Now we will consider the dynamics of consumer and producer welfares in different fishery demand scenarios, i.e., changing parameter *a* in Eq (5).

#### *a* = 1, i.e., demand remains unchanged

With the expansion of aquaculture, the substitute effect might offset the demand increase due to either growing population, improving income or changing preferences. For ease of analysis, we start with the assumption that the demand for capture products remains unchanged, i.e., *a* = 1.

**Fig 4:**
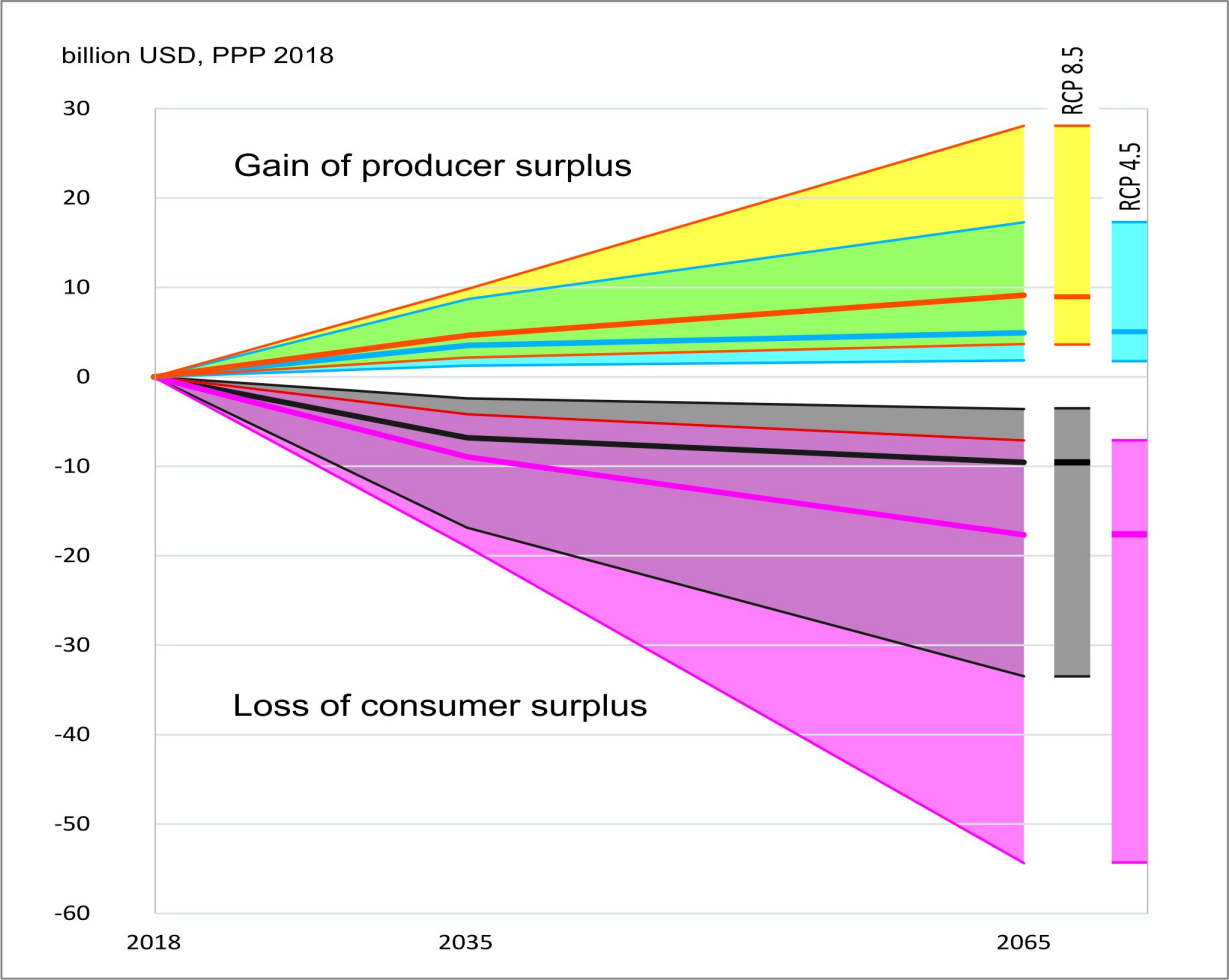
The welfare changes of fishery consumers and producers due to climate change by 2035 and 2065, relative to 2018.

Fig 4 presents the welfare changes of fishery consumers and producers due to climate change by 2035 and 2065, relative to 2018. The social discounted rate of 2% per year is applied as suggested by Drupp, Freeman [83] for the climate change context.

Fig 4 shows that producer surplus is positive and consumer surplus is negative in all RCPs. Consumer loss is from 7 to 9 billion USD per year at the mean projection of RCPs 4.5 and 8.5. By 2065, consumer loss is from 10 to 18 billion USD.

Calculations for Eqs (8) and (11) show that the capture reduction leads to a double price increase in 2035 and 3–5 times in 2065 compared to 2018. Fisher profit increases 1.7–1.9 times in 2035 and 2.7–4.2 times in 2065, depending on climate change scenarios. As depicted in Fig 4, by 2035, producer gain is from 3.5 to 4.5 billion USD per year in RCPs 4.5 – 8.5. This amount equals 30%–40% of the total capture value in 2018, approximately 12 billion USD in PPP [3]. By 2065, the gain is from 5 to 9 billion USD in RCPs 4.5 – 8.5.

Since the consumer loss is higher than producer gain, the net social loss occurs, from 3.3 to 4.3 billion USD in 2035 and 4.5 to 8.5 billion USD in 2065. All the currency values are fixed in 2018, in which 1 USD = 7465 VND in PPP (World Bank databank).

#### *b < a <* 1, i.e., relative demand reduction is less than supply reduction

Aquaculture production is proliferating in Vietnam and worldwide [2]. In both cases where farmed fish has the same or substitute value for wild fish, the expansion of aquaculture shifts consumption from wild to farmed fish. Despite the growing population and improving income, the fishery demand may decrease due to the substitution effect of aquaculture expansion.

From Eq (8):

$$\frac{P_{1}}{P_{0}}={\left(\frac{a}{b}\right)}^{\frac{1}{\varepsilon_{S}-\varepsilon_{D}}}>1\;since\;a>b\to P_{1}>P_{0}$$ (15)

From both Eqs (10) and (15):

$$\frac{{CS}_{1}}{{CS}_{0}}=a\left(1-\frac{P_{1}^{1+\varepsilon_{D}}{-P}_{0}^{1+\varepsilon_{D}}}{P_{max}^{1+\varepsilon_{D}}{-P}_{0}^{1+\varepsilon_{D}}}\right)<a<1$$ (16)

Eq (16) indicates that consumers suffer from loss due to a price increase.

From Eq (12):

$$\frac{PS_{1}}{PS_{0}}=a^{\frac{\left(1+\varepsilon_{S}\right)}{\varepsilon_{S}-\varepsilon_{D}}}b^{\frac{\left(-1-\varepsilon_{D}\right)}{\varepsilon_{S}-\varepsilon_{D}}}$$ (12)

$$\frac{{PS}_{1}}{{PS}_{0}}<1↔a^{\frac{\left(1+\varepsilon_{S}\right)}{\varepsilon_{S}-\varepsilon_{D}}}b^{\frac{\left(-1-\varepsilon_{D}\right)}{\varepsilon_{S}-\varepsilon_{D}}}<1↔a<b^{\frac{1+\varepsilon_{D}}{\varepsilon_{S}-\varepsilon_{D}}}$$ (17)

Eq (17) indicates that producers will get a profit loss when the demand reduces more than a threshold. Sensitivity analysis with changing the value of *a* indicates that if the demand for wild fish decreases by 70%–80% of the supply reduction, producers start to suffer from loss of profit.

#### *a = b <* 1, i.e., relative demand reduction equals supply reduction

If the decrease in demand equals the decrease in supply relative to 2018, i.e., *a* = *b*, then, from Eq (8):

$$\begin{array}{l} \frac{P_{1}}{P_{0}}={\left(\frac{a}{b}\right)}^{\frac{1}{\varepsilon_{S}-\varepsilon_{D}}}=1\\ \mathrm{or}\;P_{1}=P_{0},\;\mathrm{price}\;\mathrm{remains}\;\mathrm{unchanged}. \end{array}$$

From Eq (10):

$$\frac{{CS}_{1}}{{CS}_{0}}=a\left(1-\frac{P_{1}^{1+\varepsilon_{D}}{-P}_{0}^{1+\varepsilon_{D}}}{P_{max}^{1+\varepsilon_{D}}{-P}_{0}^{1+\varepsilon_{D}}}\right)=a<1$$ (18)

From Eq (12):

$$\frac{{PS}_{1}}{{PS}_{0}}=a^{\frac{\left(1+\varepsilon_{S}\right)}{\varepsilon_{S}-\varepsilon_{D}}}b^{\frac{\left(-1-\varepsilon_{D}\right)}{\varepsilon_{S}-\varepsilon_{D}}}=b^{\frac{\left(1+\varepsilon_{S}\right)}{\varepsilon_{S}-\varepsilon_{D}}}b^{\frac{\left(-1-\varepsilon_{D}\right)}{\varepsilon_{S}-\varepsilon_{D}}}=b<1$$ (19)

Eqs (18) and (19) depict that consumers and producers get losses by *b* relative to 2018 due to the reduction in production quantity while the price remains constant. In this case, the producer profit will likely reduce 20% to 24% by 2035 and 35% to 46% by 2065. The loss of fisher profit is from 1.0 to 1.3 billion USD (PPP, 2018) by 2035 and 2065, equal to 9%–11% of total capture value in 2018.

### *a < b <* 1, i.e., relative demand reduction is higher than supply reduction

From Eq (8):

$$\begin{array}{l} \frac{P_{1}}{P_{0}}={\left(\frac{a}{b}\right)}^{\frac{1}{\varepsilon_{S}-\varepsilon_{D}}}<1\\ \mathrm{or}\;P_{1}<P_{0},\;\mathrm{fishery}\;\mathrm{price}\;\mathrm{decreases}. \end{array}$$

From Eq (10):

$$\frac{{CS}_{1}}{{CS}_{0}}=a\left(1-\frac{P_{1}^{1+\varepsilon_{D}}{-P}_{0}^{1+\varepsilon_{D}}}{P_{max}^{1+\varepsilon_{D}}{-P}_{0}^{1+\varepsilon_{D}}}\right)\approx a<1$$ (20)

From Eq (12):

$$\frac{{PS}_{1}}{{PS}_{0}}=a^{\frac{\left(1+\varepsilon_{S}\right)}{\varepsilon_{S}-\varepsilon_{D}}}b^{\frac{\left(-1-\varepsilon_{D}\right)}{\varepsilon_{S}-\varepsilon_{D}}}<b^{\frac{\left(1+\varepsilon_{S}\right)}{\varepsilon_{S}-\varepsilon_{D}}}b^{\frac{\left(-1-\varepsilon_{D}\right)}{\varepsilon_{S}-\varepsilon_{D}}}=b<1$$ (21)

Eqs (20) and (21) show that consumers and producers get losses.

### *a >* 1, i.e., demand increases

From Eq (8):

$$\begin{array}{l} \frac{P_{1}}{P_{0}}={\left(\frac{a}{b}\right)}^{\frac{1}{\varepsilon_{S}-\varepsilon_{D}}}>1\\ \mathrm{or}\;P_{1}>P_{0},\;\mathrm{fishery}\;\mathrm{price}\;\mathrm{increases}. \end{array}$$

From Eq (12), we have:

$$\frac{{PS}_{1}}{{PS}_{0}}=a^{\frac{\left(1+\varepsilon_{S}\right)}{\varepsilon_{S}-\varepsilon_{D}}}b^{\frac{\left(-1-\varepsilon_{D}\right)}{\varepsilon_{S}-\varepsilon_{D}}}>1↔a>b^{\frac{1+\varepsilon_{D}}{\varepsilon_{S}-\varepsilon_{D}}}$$ (22)

Eq (22) is correct since $a>1>b^{\frac{1+\varepsilon_{D}}{\varepsilon_{S}-\varepsilon_{D}}}$.

Therefore, when demand increases, producer surplus increases.

We recall Eq (10):

$$\frac{CS_{1}}{CS_{0}}=a(1-\frac{P_{1}^{1+\varepsilon_{D}}-P_{0}^{1+\varepsilon_{D}}}{P_{{max}^{1+\varepsilon_{D}}}-P_{0}^{1+\varepsilon_{D}}})$$ (10)

Eq (10) is increasing in *a*. When *a* = 1, $\frac{{CS}_{1}}{{CS}_{0}}<1$. When *a* is large enough, then $\frac{{CS}_{1}}{{CS}_{0}}>1$, which means that consumers start to gain if their demand surpasses a certain threshold.

Table 5 summarises the dynamics of producer and consumer welfares, with respect to an increase in *a* while *b* keeps constant from the previous results.

**Table 5:** The dynamics of producer and consumer welfares under climate change.

	*a < b <* 1	*a = b <* 1	*b < a <* 1	*a =* 1	*a >* 1
Consumer surplus	–	–	–	–	– then +
Producer surplus	–	–	– then +	+	+
Social surplus	–	–	–	–	– then +

## Discussions and conclusions

The research results confirm the adverse impact of climate change on catch yield. Temperature rise is the primary cause of the declining production since it triggers tropical fish to migrate to more comfortable areas and the rest to reduce their sizes and stocks [4, 7, 54, 56, 58, 59]. The impact of temperature increases with time and severity of climate change scenarios. The capture yield will reduce 35% from now to mid-century under the RCP4.5. This result is close to Cheung et al.’s (2010) projection that the catch potential in the Pacific Ocean in 10N–30N will decrease up to 30% until 2055 under the B1 (equivalent to RCP4.5) scenario. The worst scenario predicts that there will be no capture by the end of the century. This forecast is not surprising, as it has recently been almost no river capture in Vietnam.

The negative impacts of sea surface temperature, rainfall, typhoons, and wind speed on catch yields are also found in various literature in Vietnam [61] and other places [17, 27, 42, 47, 62–64, 71, 73, 84]. For instance, Garza-Gil, Torralba-Cano [84] report a 10% rise in temperature leading to a 1.4% annual decrease in the European sardine fishery’s profit. The total Taiwan trout population is likely to decline from 1612 to 974 if precipitation increases by 0.6 mm per day and the temperature increases by 0.9°C [73]. Meynecke, Grubert [64] indicate that a high SOI value enhances the productivity of giant mud crab *(Scylla Serrata)* catches in Northern Australia. Alnafissa, Kotb [47] realize an increase of 10% in the wind speed heading to a decrease of 6.8% in fish production in Saudi Arabia.

The welfare impacts of climate change on producers and consumers depend significantly on the change in wild fish demand.

If the demand curve remains unchanged, then the drop in supply induces fishery price increase, creating tremendous loss to consumers, from 7 to 9 billion USD by 2035 and from 10 to 18 billion USD in 2065 (PPP, 2018). If the demand decreases, mainly due to the substitution effect of the aquaculture expansion, consumers also suffer from loss. If the demand increases, primarily owing to the growing population and improving income, consumers may benefit if the demand surpasses a certain level. However, the latter case is not likely to happen since aquaculture is escalating rapidly, and consumption is more likely to shift from wild to farmed fish.

In contradiction to many concerns that climate change will affect producers adversely as it reduces capture revenues [27, 29, 36, 85], the research results find that fishers gain more than lose thanks to the price upsurge if the demand for wild fish remains unchanged. If there is no price increase, i.e., demand for wild fish decreases as farmed fish is more available, the fisher’s profit reduces 20%–24% of their profit in 2018 by 2035 and 35%–46% by 2065. The loss of profit is from 1.0 to 1.3 billion USD (PPP, 2018). Since the consumer preferences on wild fish and farmed fish are different for individuals, it is not likely that farmed fish can fully replace wild fish. Hence, fishers have more chance to benefit than lose.

These findings suggest that although climate change hinders fishing production, fisher’s welfare is more likely to increase, and consumer welfare is more likely to decrease.

In Vietnam, the capture fishery sector is a pro-poor economic sector. The fishery policies usually aim to maximize social welfare, helping people escape poverty. Therefore, wild fish is open access, leading to the spread of small-scale fishers along the seaside, escalating stress on nearshore fish stock. No specific actions limiting small-scale capture have been implemented in the Vietnam fishery sector. The application of quotas for inshore and offshore fishing vessels, newly introduced since 2019, is controversial and faces many obstacles, especially from fishers. The research findings imply that limiting capture effort do not harm fishers’ welfare in the long run.

The Vietnam Fisheries Development Strategy for 2021-2030 [31] sets its goals to develop aquaculture, especially marine species, as an adaptation action for dealing with the reduction of capture. However, saline aquaculture is often achieved by over-fishing wild juveniles and stocks to serve as food for cultivated species [86]. Therefore, the impact of expanding aquaculture on capture remains ambiguous. Furthermore, the change in wild fish demand remains uncertain. More research is needed to understand how aquaculture expansion could impact capture producers’ income, employment, and profits.

## Supporting information

S1 Data 1
(RAR)
